# Coping strategies in patients with brain neoplasms: clinical psychological differences and adaptive potential

**DOI:** 10.1007/s11060-026-05849-0

**Published:** 2026-10-31

**Authors:** Kamilla Kholmuradova, Alexandra Maslennikova

**Affiliations:** 1 Europäische Fernhochschule Hamburg (Euro-FH), University of Applied Sciences, Hamburg, Germany; 2 Mental Health Hospital N°1, named after N.A Alexeev, Moscow, Russia

**Keywords:** Brain tumors, Meningiomas, Glioneuronal tumors, Coping strategies, Meaning-focused coping, Quality of life, Posttraumatic growth, Anxiety, Depression

## Abstract

**Background:**

Coping behavior is an important component of psychological adaptation in patients with brain neoplasms, whose illness may involve neurological deficits, cognitive impairment, prognostic uncertainty, and threats to autonomy and personal identity. However, evidence on coping patterns across different types of brain tumors and their relationships with quality of life, affective state, and post-traumatic growth remains limited.

**Aim:**

To examine the characteristics of coping behavior in patients with brain neoplasms and to assess the associations between specific coping strategies and quality of life, affective state, and post-traumatic growth.

**Materials and methods:**

The study included 102 patients with brain neoplasms treated in neurosurgical departments in the Republic of Uzbekistan (Mage = 42.5, SD = 15.35; 52 women and 50 men). Patients were divided into three groups according to the histological type of neoplasm: meningiomas (*n* = 34), glioneuronal tumors (*n* = 35), and other neoplasms (*n* = 33). Coping behavior was assessed using the brief version of the COPE-30 questionnaire and an additional Meaning-Focused Coping Scale. Quality of life was assessed using the EORTC QLQ-C30 questionnaire and its brain cancer-specific module, QLQ-BN20. Anxiety and depression were assessed using the Hospital Anxiety and Depression Scale (HADS), and post-traumatic growth was assessed using the Post-Traumatic Growth Inventory (PTGI).

**Results:**

Active coping was the most frequently used strategy in the overall sample, whereas mental disengagement was the least frequently used. Patients with meningiomas more frequently reported active coping, planning, and meaning-focused coping, whereas religious coping and humor were relatively more common among patients with glioneuronal tumors. Meaning-focused coping was positively associated with post-traumatic growth, satisfaction with health, and emotional functioning, and negatively associated with depressive symptoms. Its association with anxiety was not statistically significant.

**Discussion:**

The findings suggest that coping behavior represents an important component of the subjective experience of illness in patients with brain tumors. In the present study, the greatest adaptive potential was observed for coping strategies involving active patient engagement in managing the illness and reappraising its personal meaning.

**Conclusion:**

Assessment of coping strategies in patients with brain neoplasms may have clinical relevance for psychological support and could inform the development of differentiated psychosocial intervention programs.

**Supplementary Information:**

The online version contains supplementary material available at https://doi.org/10.1007/s11060-026-05849-0.

## Background

Patient involvement in therapeutic procedures, psychological adaptation, and acceptance of the diagnosis play a crucial role in the treatment of cancer. Taking into account individual psychological differences in coping strategies across different cancer diagnoses may help psycho-oncologists prepare patients for acceptance of their diagnosis and foster a more positive attitude toward treatment.

Psychological adaptation in patients with brain neoplasms is determined not only by the somatic burden of the disease but also by the combination of neurological deficits, cognitive impairments, prognostic uncertainty, and a pronounced existential threat. Unlike many other types of cancer, brain tumors affect functions directly related to autonomy, speech, memory, and personal identity, making coping behavior particularly clinically relevant in this patient population. According to contemporary research in psycho-oncology and neuro-oncology, patients with gliomas and other brain tumors experience high rates of unmet supportive care needs, anxiety and depressive symptoms, and impaired quality of life [1, 2]. At the same time, the literature specifically addressing coping strategies in neuro-oncology remains limited and heterogeneous with regard to sample characteristics, disease types, and psychometric instruments used [3–5].

In the present study, histological tumor type was selected as the basis for classification, based on the assumption that patients may demonstrate different coping profiles depending on the biological characteristics of the brain tumor, particularly when comparing malignant tumors (e.g., gliomas) with benign tumors. To date, relatively few studies have examined responses to diagnosis, illness representations, and adaptive strategies in patients with brain tumors as a function of tumor type and malignancy [6, 7]. The practical significance of the present study lies in improving psychological support for patients with malignant neoplasms. Based on the findings, it may be possible to develop a psychological support protocol aimed at stabilizing patients’ psychological well-being, improving quality of life, and enhancing their engagement in treatment [8, 9].

Available evidence suggests that more active and less avoidant coping styles are more frequently associated with better psychological functioning, whereas avoidance is associated with greater distress and poorer quality-of-life outcomes [10–12]. In addition, meaning-making plays an important role in psychological adaptation: several studies of patients with cancer have shown that finding or reconstructing meaning in the experience of illness is associated with post-traumatic growth and more favorable adaptation [13–16]. Interview-based studies examining the acceptance of a malignant cancer diagnosis indicate that the experience of loss of functional capacity is a central concern. In patients with brain tumors, these patterns require separate investigation because the subjective experience of illness is shaped not only by the threat to life but also by the risk of cognitive, speech, and functional impairment. The type of diagnosis is therefore an important factor to consider, as patients may perceive and adapt to a diagnosis of a benign tumor more positively than to a malignant one [17–19].

From the perspective of clinical psychology, coping behavior can be regarded as one of the key behavioral manifestations of illness representation. Its analysis makes it possible to assess the extent to which patients adopt an active position toward treatment, seek social support, engage in avoidance or denial, rely on religious explanations, or attempt to integrate the illness into their broader system of personal meaning [16, 20]. This approach is particularly relevant when comparing different histological groups, as the subjective “illness narrative,” perceived prognosis, rate of disease progression, and clinical characteristics of the tumor may be associated with differences in psychological adaptation.

Against this background, the present study aimed to characterize coping strategies in patients with different types of brain neoplasms, with a particular focus on malignant and benign tumors, and to examine the associations between specific coping strategies and quality of life, affective state, and post-traumatic growth [21].

## Methods

### Participants

The study included 102 patients with brain neoplasms who were treated at the neurosurgical departments of the Republican Scientific Center of Neurosurgery of the Ministry of Health of the Republic of Uzbekistan between 2025 and 2026. Patients were recruited from the outpatient population receiving treatment during the study period. According to the histological type of neoplasm, patients were divided into three groups: meningiomas (*n* = 34), glioneuronal tumors (*n* = 35), and other neoplasms (*n* = 33).

A preliminary screening of cognitive status was performed. Patients with severe cognitive impairment were excluded from the main psychological analysis (*n* = 12).

The inclusion criteria were: a primary diagnosis or recurrence of a brain neoplasm; age between 18 and 78 years (M ± SD = 42.5 ± 15.35); absence of significant comorbid pathology; and a Montreal Cognitive Assessment (MoCA) score ranging from 10 to 30. Participants scoring 10–21 on the MoCA were retained in the study. The exclusion criteria were a MoCA score ranging from 0 to 9, a history of traumatic brain injury, stroke, epilepsy, or chronic neurological disorders, as well as age below 18 years.

Clinical recruitment included patients from Department No. 4 of Neuro-Oncology and Department No. 5 of Skull Base Tumors. The distribution of patients by department, treatment phase, and diagnostic status is reported in the Results section.

During the assessment period, none of the patients received chemotherapy, radiotherapy, or radiation therapy. Almost all patients received medication for cerebral edema (dexamethasone) and/or anticonvulsant treatment (carbamazepine). This relatively homogeneous treatment profile was intended to reduce, but could not eliminate, treatment-related confounding of psychological measures.

Detailed patient characteristics are reported in the Results section and in Supplementary Materials 1 and 2.

### Psychometric measures

Coping behavior, quality of life, affective symptoms, post-traumatic growth, and cognitive status were assessed with standardized self-report instruments and the MoCA. The selected instruments were chosen because their established constructs correspond to the study objectives: coping strategies, illness-related meaning-making, health-related quality of life, affective symptoms, post-traumatic growth, and cognitive screening.

Coping behavior was assessed using the 30-item short Russian-language version of the COPE questionnaire (COPE-30), which assesses 15 coping strategies, including active coping, planning, emotional social support, avoidance, denial, mental disengagement, religious coping, humor, and substance/sedative use [3]. Meaning-focused coping was assessed with the Meaning-Focused Coping Scale, which captures efforts to find, preserve, or reconstruct personal meaning in response to a stressful illness experience [22].

Health-related quality of life was assessed with the 30-item EORTC QLQ-C30, which includes a global health status/quality-of-life scale, five functioning scales, and symptom scales/items, together with the 20-item EORTC QLQ-BN20 brain cancer module assessing brain-tumor-specific symptoms and concerns [17]. Anxiety and depression were measured with the 14-item Hospital Anxiety and Depression Scale (HADS), comprising two seven-item subscales [5]. Post-traumatic growth was measured with the 21-item Post-Traumatic Growth Inventory (PTGI), which assesses positive changes across five domains after a traumatic event [6]. The 30-point MoCA was used as a cognitive screening measure covering executive, attentional, memory, language, visuospatial, and orientation functions.

### Statistical analysis

All tests were two-sided, with a statistical significance threshold of *p* < 0.05. Distributional assumptions were assessed using the Shapiro–Wilk test. Because the primary coping-score distributions departed from normality, between-group comparisons across the three diagnostic groups were performed using the Kruskal–Wallis H test. When an omnibus test was significant, post hoc pairwise comparisons were evaluated using the Bonferroni correction. Between-sex comparisons were performed using the Mann–Whitney U test. Associations between coping strategies and HADS scores were examined using Pearson correlation coefficients, as reported in Table 2. For analyses involving multiple comparisons, the Bonferroni-adjusted threshold was applied.

**Table 1:** Intergroup differences in coping strategies used (Kruskal–Wallis H-test)

Coping scales	p	Group mean ranks (MR)
Mental disengagement from the problem	< 0.001	Gr2 (4.0) > Gr1 (2.5)
Avoidance	0.003	Gr3 (3.3) > Gr1 (2.5)
Active coping	< 0.001	Gr1 (6.7) > Gr2 (3.7)
Meaning-focused coping	< 0.001	Gr1 (47.8) > Gr3 (32.5) > Gr2 (22.9)
Use of sedatives	0.034	Gr2 (3.6) > Gr1 (2.5)

Note* — patients with meningiomas (Gr1), patients with glial tumors (Gr2), patients with other neoplasms (Gr3)

### Ethical considerations

The study was conducted in accordance with the principles of the Declaration of Helsinki and was approved by the Ethics Committee of Samarkand State Medical University (Protocol No. 010, approved on January 26, 2026). All participants provided written informed consent prior to participation in the study.

### Declaration of generative AI use

The authors did not use generative AI in the design, conduct, analysis, interpretation of the research, or in the writing of this manuscript. Generative AI was used solely to assist in the creation of a figure for illustrative purposes. The authors reviewed, edited, and take full responsibility for the final content of the manuscript.

## Results

The final analytical sample comprised 102 patients with brain neoplasms (Mage = 42.5 years, SD = 15.35; range, 18–78 years), including 52 women and 50 men. According to histological diagnosis, 34 patients had meningiomas, 35 had glioneuronal tumors, and 33 had other neoplasms. Sixty-four patients were recruited from Department No. 4 of Neuro-Oncology and 36 from Department No. 5 of Skull Base Tumors. At assessment, 58 patients were undergoing outpatient preoperative preparation, 38 were assessed 1–4 days after surgery, six received pharmacological treatment only because surgery was not indicated, and two were not receiving outpatient treatment. Seventy-nine patients had a primary diagnosis and 23 had recurrent neoplasms. Shapiro–Wilk tests indicated non-normal score distributions for the primary coping analyses (*p* < 0.05); nonparametric group comparisons were therefore used.

The Kruskal–Wallis H test identified statistically significant diagnostic-group differences in five coping strategies (Table 1). Patients with glioneuronal tumors reported greater mental disengagement from the problem than patients with meningiomas (*p* < 0.001; mean ranks 4.0 vs. 2.5). Avoidance was more pronounced in the other-neoplasms group than in the meningioma group (*p* = 0.003; mean ranks 3.3 vs. 2.5). In contrast, active coping was higher among patients with meningiomas than among patients with glioneuronal tumors (*p* < 0.001; mean ranks 6.7 vs. 3.7). Meaning-focused coping showed an ordered pattern, with the highest mean rank in the meningioma group (47.8), followed by the other-neoplasms group (32.5) and the glioneuronal-tumor group (22.9; *p* < 0.001). Use of sedatives was higher in the glioneuronal-tumor group than in the meningioma group (*p* = 0.034; mean ranks 3.6 vs. 2.5).

Between-sex comparisons identified differences in two coping scales. Use of Emotional Social Support was higher among women than men (*p* < 0.001), whereas Use of Tranquilizing Drugs was higher among men than women (*p* < 0.001).

Pearson correlation analyses examined associations between coping strategies and affective symptoms (Table 2). Lower depression scores were associated with greater denial (*r* = − 0.302, *p* = 0.004) and greater meaning-focused coping (*r* = − 0.232, *p* = 0.030). Higher anxiety scores were associated with greater mental disengagement from the problem (*r* = 0.299, *p* = 0.005) and greater use of sedatives (*r* = 0.212, *p* = 0.048), whereas active coping was inversely associated with anxiety (*r* = − 0.301, *p* = 0.005). These correlations indicate associations within this cross-sectional sample and should not be interpreted as causal effects.

**Table 2:** Associations between coping strategies and anxiety and depression indicators (Pearson correlation coefficient)

HADS scales	Coping strategy	Pearson’s *r*	*p*-value
Depression level	Denial	−0.302	0.004
Depression level	Meaning-focused coping	−0.232	0.030
Anxiety level	Mental disengagement from the problem	0.299	0.005
Anxiety level	Active coping	−0.301	0.005
Anxiety level	Use of “sedatives”	0.212	0.048

**Fig. 1:**
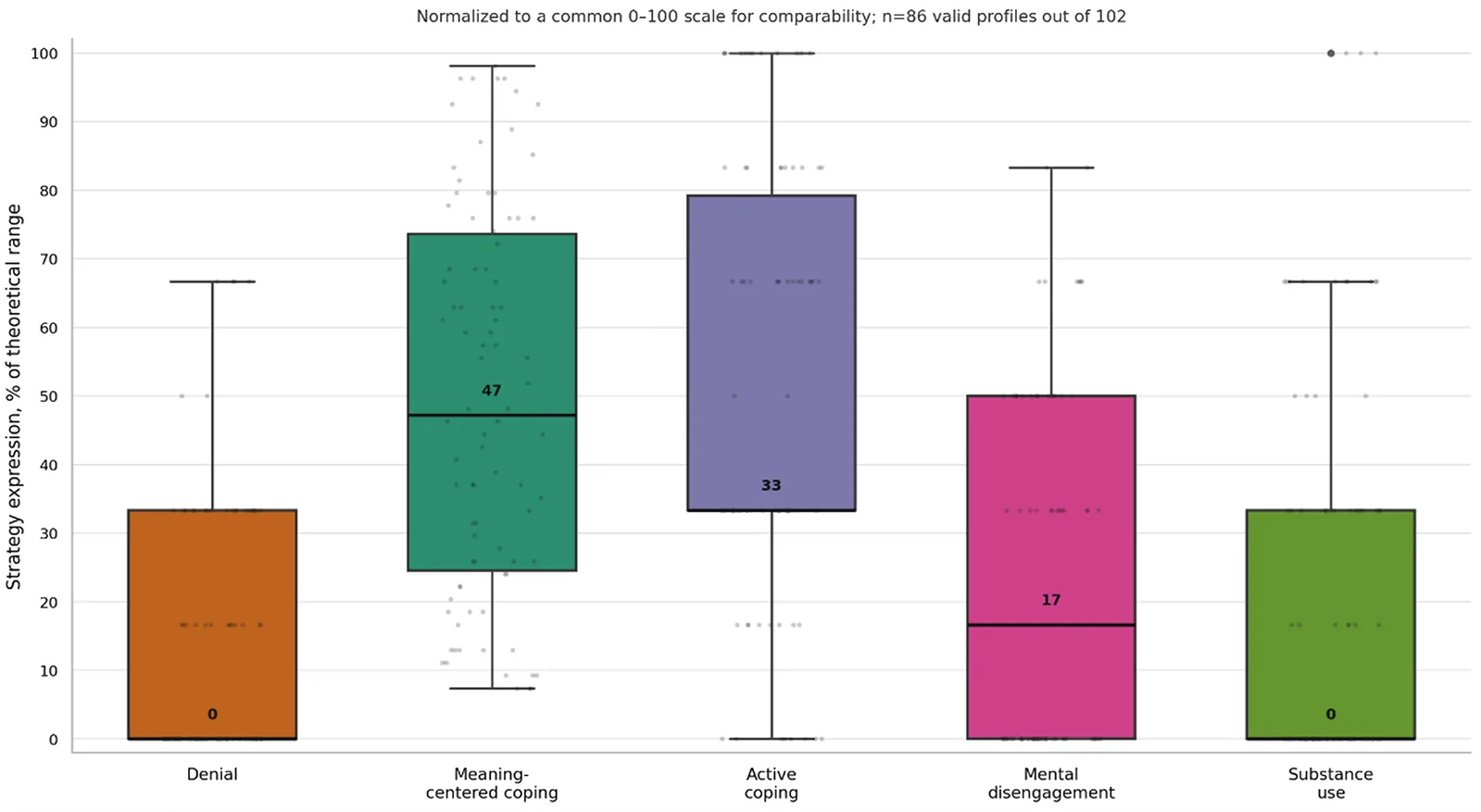
Coping strategy expression in the sample

The distribution of coping strategy scores showed considerable variation across the assessed strategies. Active coping and meaning-focused coping demonstrated the highest median scores (33 and 47, respectively), whereas mental disengagement had a lower median score of 17. Denial and substance use showed the lowest median scores, both equal to 0. The widest score ranges were observed for active and meaning-focused coping, indicating substantial individual variability in the expression of these strategies (Fig. 1).

In addition, the correlation analysis showed that meaning-focused coping was associated with higher levels of post-traumatic growth (*r* = 0.58; *p* < 0.001) and was positively associated with specific aspects of quality of life, particularly health satisfaction (*r* = 0.46; *p* < 0.001) and emotional functioning (*r* = 0.44; *p* < 0.001).

## Discussion

In the overall sample, active coping was the most frequently used strategy, whereas mental disengagement was relatively uncommon. This finding is of particular interest, as it partially contrasts with some findings in psycho-oncology, where avoidance-oriented coping may play a more prominent role in patients with severe illnesses [23–25]. In patients with brain neoplasms, the predominance of active coping may be related to the high subjective significance of the threat of losing cognitive and functional autonomy.

The diagnostic-group comparison should be interpreted at the level of the specific strategies that differed. Patients with meningiomas showed higher active coping and meaning-focused coping than patients with glioneuronal tumors; meaning-focused coping was also higher than in the other-neoplasms group. In contrast, patients with glioneuronal tumors showed more mental disengagement and greater use of sedatives than patients with meningiomas, while avoidance was more pronounced in the other-neoplasms group than in the meningioma group. These patterns are compatible with literature describing substantial psychosocial burden and supportive-care needs in patients with primary brain tumors [7, 26, 27]. They do not, however, establish that a particular histological diagnosis directly causes a particular coping response.

Meaning-focused coping is of particular interest because it may represent a psychological resource for adaptation to illness. Unlike purely instrumental coping, it involves reappraising the illness experience and integrating it into a broader personal system of meaning [13, 22]. In the present sample, higher meaning-focused coping was associated with greater post-traumatic growth and with more favorable ratings of health satisfaction and emotional functioning. This pattern is consistent with evidence that meaning-making and positive reappraisal may be related to post-traumatic growth and quality of life in cancer populations [1, 2, 20, 21]. In a study of patients with low-grade gliomas, post-traumatic growth was also associated with quality of life, with the association varying according to post-traumatic stress symptoms and avoidant coping [28]. Because the design was cross-sectional, the direction of these associations cannot be determined: better adjustment may facilitate meaning-making, meaning-making may facilitate adjustment, or both may be influenced by unmeasured clinical and social factors.

The affective-symptom correlations add a more differentiated picture. Active coping was associated with lower anxiety, whereas mental disengagement and use of sedatives were associated with higher anxiety. Denial and meaning-focused coping were associated with lower depressive symptoms. These findings should be interpreted cautiously. In particular, lower depressive symptoms in patients reporting denial do not demonstrate that denial is protective; denial may have different short- and long-term implications depending on disease stage, illness awareness, treatment demands, and functional status. Existing psycho-oncology literature similarly cautions against classifying coping strategies as uniformly adaptive or maladaptive outside their clinical context [23, 25, 29].

### Clinical implications

A profile of coping strategies and the subjective perception of the illness provides both the physician and the accompanying psychologist with information on the type of motivation to use when engaging the patient in treatment procedures. This enables a personalized approach to fostering a positive and active attitude toward treatment.

### Study limitations

The study has several limitations. Medication use was not controlled for, which may have influenced on well-being and self-report, quality of life and questionnaire scores as a result. The study was conducted at a single site in Uzbekistan, and cross-cultural validation is necessary to establish generalizability. Finally, lack of control over tumor localization and associated cognitive, motor, and/or emotional deficits, which could also have influenced the ability to provide self-assessments when completing questionnaires.

## Conclusion

In patients with brain neoplasms, coping behavior represents a clinically relevant component of psychological adaptation to illness. In the present sample, active coping was the most frequently used strategy. The between-group analysis showed that patients with meningiomas more frequently used active coping, planning, and meaning-focused coping, whereas religious coping and humor were relatively more common among patients with glioneuronal tumors. Meaning-focused coping was associated with higher levels of post-traumatic growth, more favorable subjective evaluations of health and emotional functioning, and slightly lower levels of depressive symptoms. These findings suggest that the assessment of coping strategies may serve as an important component of clinical psychological assessment and provide a basis for developing differentiated psychological support programs for patients with neuro-oncological conditions.

## Supplementary Information

Below is the link to the electronic supplementary material.

Supplementary Material 1

## Data Availability

No datasets were generated or analysed during the current study.

## References

[1] Adamkovič M, Fedáková D, Kentoš M et al (2022) Relationships between satisfaction with life, posttraumatic growth, coping strategies, and resilience in cancer survivors: a network analysis approach. Psychooncology 31(12):1913–1921. 10.1002/pon.5948 PMC9790334 35524705

[2] Senger AR, Ratcliff CG, Semelsberger RK, Chaoul MA, Cohen L (2023) The role of coping and posttraumatic stress in fostering posttraumatic growth and quality of life among women with breast cancer. J Clin Psychol Med Settings 30(4):712–725. 10.1007/s10880-023-09945-x PMC11771815 37803095

[3] Odintsova MA, Radchikova NP, Aleksandrova LA. COPE-30: psychometric properties of the short version of the Russian-language inventory for coping strategies evaluation. Moscow Univ Psychol Bull. 2022;(4):247–75. 10.11621/vsp.2022.04.11.

[4] Smith BW, Dalen J, Wiggins K, Tooley E, Christopher P, Bernard J (2008) The brief resilience scale: assessing the ability to bounce back. Int J Behav Med 15(3):194–200 10.1080/10705500802222972 18696313

[5] Zigmond AS, Snaith RP (1983) The Hospital Anxiety and Depression Scale. Acta Psychiatr Scand 67(6):361–370 10.1111/j.1600-0447.1983.tb09716.x 6880820

[6] Tedeschi RG, Calhoun LG (1996) The Posttraumatic Growth Inventory: measuring the positive legacy of trauma. J Trauma Stress 9(3):455–471 10.1007/BF02103658 8827649

[7] Guariglia L, Ieraci S, Villani V, Tanzilli A, Benincasa D, Sperati F, Terrenato I, Pace A (2021) Coping Style in Glioma Patients and Their Caregiver: Evaluation During Disease Trajectory. Front Neurol 12:709132. 10.3389/fneur.2021.709132 PMC8440902 34539556

[8] Jim HS, Jacobsen PB (2008) Posttraumatic stress and posttraumatic growth in cancer survivorship: a review. Cancer J 14(6):414–419 10.1097/PPO.0b013e31818d8963 19060607

[9] Diener E, Emmons RA, Larsen RJ, Griffin S (1985) The Satisfaction With Life Scale. J Pers Assess 49(1):71–75 10.1207/s15327752jpa4901_13 16367493

[10] Watson M, Greer S, Young J, Inayat Q, Burgess C, Robertson B (1988) Development of a questionnaire measure of adjustment to cancer: the MAC scale. Psychol Med 18(1):203–209 10.1017/s0033291700002026 3363039

[11] Cordova MJ, Giese-Davis J, Golant M et al (2007) Breast cancer as trauma: posttraumatic stress and posttraumatic growth. J Clin Psychol Med Settings 14(4):308–319

[12] Bozo Ö, Gündoğdu E, Büyükşahin A (2017) The mediating role of posttraumatic growth and resilience in the relationship between social support and quality of life in cancer patients. Psychooncology 26(10):1535–1542

[13] Park CL (2010) Making sense of the meaning literature: an integrative review of meaning making and its effects on adjustment to stressful life events. Psychol Bull 136(2):257–301 10.1037/a0018301 20192563

[14] Casellas-Grau A, Font A, Vives J (2014) Positive psychology interventions in breast cancer: a systematic review. Psychooncology 23(1):9–19 10.1002/pon.3353 23897834

[15] Singer S, Dieng S, Wesselmann S (2013) Psycho-oncological care in certified cancer centres–a nationwide analysis in Germany. Psychooncology 22(6):1435–1437. 10.1002/pon.3145 22855347

[16] Meyerowitz BE, Kurita K, D’Orazio LM (2001) The psychological and social impact of cancer. In: Baum A, Anderson BL (eds). Psychosocial Interventions for Cancer. American Psychological Association, Washington, DC, pp 1–14

[17] Taphoorn MJB, Claassens L, Aaronson NK et al (2010) An international validation study of the EORTC brain cancer module (EORTC QLQ-BN20) for assessing health-related quality of life and symptoms in brain cancer patients. Eur J Cancer 46(6):1033–1040. 10.1016/j.ejca.2010.01.012 20181476

[18] Kershaw T, Northouse L, Kritpracha C, Schafenacker A, Mood D (2004) Coping strategies and quality of life in women with advanced breast cancer and their family caregivers. Psychol Health 19(2):139–155

[19] Lutgendorf SK, Andersen BL (2009) Biobehavioral aspects of cancer: stress and the immune system. In: Miller SM, Bowen DJ, Croyle RT, Rowland JH (eds). Handbook of Cancer Control and Behavioral Science. American Psychological Association, Washington, DC, pp 89–106

[20] Schroevers MJ, Kraaij V, Garnefski N (2011) Cancer patients’ experience of positive and negative changes due to the illness: relationships with psychological well-being, coping, and goal reengagement. Psychooncology 20(2):165–172 10.1002/pon.1718 20217657

[21] Sears SR, Stanton AL, Danoff-Burg S (2003) The yellow brick road and the emerald city: benefit finding, positive reappraisal coping, and posttraumatic growth in women with early-stage breast cancer. Health Psychol 22(5):487–497 10.1037/0278-6133.22.5.487 14570532

[22] Leontiev DA, Rasskazova EI, Taranenko OA (2025) Meaning-Centered Coping as a Target of Psychological Assessment. Konsultativnaya Psikhologiya i Psikhoterapiya 33(2):171–190 In Russ. 10.17759/cpp.2025330208

[23] Nipp RD, El-Jawahri A, Fishbein JN et al (2016) The relationship between coping strategies, quality of life, and mood in patients with incurable cancer. Cancer 122(13):2110–2116. 10.1002/cncr.30025 PMC5160928 27089045

[24] Mor V, Guadagnoli E (1988) Quality of life measurement: a psychometric test of the Functional Living Index-Cancer (FLIC). J Clin Oncol 6(5):881–888

[25] Faller H, Schuler M, Richard M, Heckl U, Weis J, Küffner R (2013) Effects of psycho-oncologic interventions on emotional distress and quality of life in adult patients with cancer: systematic review and meta-analysis. J Clin Oncol 31(6):782–793 10.1200/JCO.2011.40.8922 23319686

[26] Catt S, Chalmers A, Fallowfield L (2008) Psychosocial and supportive-care needs in high-grade glioma. Lancet Oncol 9(9):884–891. 10.1016/S1470-2045(08)70230-4 18760244

[27] Ford E, Catt S, Chalmers A, Fallowfield L (2012) Systematic review of supportive care needs in patients with primary malignant brain tumors. Neuro Oncol 14(4):392–404. 10.1093/neuonc/nor229 PMC3309849 22307475

[28] Chen Q, Xu Y, Yang L et al (2019) Are Posttraumatic Stress Symptoms and Avoidant Coping Inhibitory Factors? The Association Between Posttraumatic Growth and Quality of Life Among Low-Grade Gliomas Patients in China. Front Psychol 10:330. 10.3389/fpsyg.2019.00330 PMC6401985 30873074

[29] Stanton AL, Revenson TA, Tennen H (2007) Health psychology: psychological adjustment to chronic disease. Annu Rev Psychol 58:565–592 10.1146/annurev.psych.58.110405.085615 16930096

