# Supplementary material for: Coping strategies in patients with brain neoplasms: clinical psychological differences and adaptive potential
Source: J Neurooncol. 2026 Oct 31;180(2):34. doi: 10.1007/s11060-026-05849-0 (PMC13723449; doi:10.1007/s11060-026-05849-0)
Supplement: Supplementary file 1 [file 11060_2026_5849_MOESM1_ESM.docx]

Supplementary Materials

*Table 1. Characteristics of the Respondents*

| **Respondent Characteristics** | **Value** |
| --- | --- |
| Total number of participants | 102 |
| Number of women | 52 |
| Number of men | 50 |
| Minimum age | 18 |
| Maximum age | 78 |
| Mean age (overall sample) | 44.15 |
| Mean age (Neuro-Oncology Department) | 47.6 |
| Mean age (Skull Base Surgery Department) | 37.7 |
| Standard deviation of age | 15.35 |
| Median age | 42.5 |
| Have a partner (married or in a civil partnership) | 46 |
| Do not have a partner (single, divorced, or widowed) | 56 |
| Do not have children | 32 |
| Have children (1 to 10) | 70 |
| Engaged in educational or employment activities | 50 |
| Not engaged in a specific occupation / homemakers / retired | 52 |

*Table 2. Tumor Localization in Patients with Intracerebral Tumors (Neuro-Oncology Department)*

| **Tumor Localization** | **Right Hemisphere** | **Left Hemisphere** | **Both Hemispheres** |
| --- | --- | --- | --- |
| Occipital region | 0 | 0 | 0 |
| Parietal region | 1 | 4 | 0 |
| Parieto-occipital region | 3 | 1 | 0 |
| Parieto-temporal region | 2 | 2 | 0 |
| Temporal region | 5 | 7 | 2 |
| Temporo-occipital region | 0 | 1 | 0 |
| Frontal region | 6 | 6 | 0 |
| Fronto-parietal region | 5 | 0 | 0 |
| Fronto-temporal region | 1 | 2 | 0 |
| Base of the cerebral hemispheres | 0 | 2 | 0 |
| Falx cerebri (various parts) | 4 | 0 | 0 |
| Ventricles | 1 | 1 | 0 |
| Multiple neoplasms | 1 | 3 | 5 |

*Table 3. Tumor Localization in Patients with Extracerebral Tumors (Skull Base Surgery Department)*

| **Tumor Localization** | **Right Side** | **Left Side** |
| --- | --- | --- |
| Anterior cranial fossa | 3 | 0 |
| Middle cranial fossa | 1 | 1 |
| Posterior cranial fossa | 1 | 0 |
| Chiasmatic-sellar region (CSR) | 10 | |
| Cerebellopontine angle | 4 | 2 |
| Cerebellar vermis | 1 | 0 |
| Cerebellar hemisphere | 0 | 1 |
| Petroclival region | 1 | |
| Diencephalic region | 3 | |
| Mixed localizations (with invasion) | 8 | |

*Table 4. Histological Types of Tumors in the Study Population*

| **Tumor Group** | **Tumor Type** | **N of Patients** |
| --- | --- | --- |
| **Meningiomas** | Meningioma | 29 |
|  | Fibrous meningioma | 5 |
|  | Angiomatous meningioma | 1 |
| **Glioneuronal tumors** | Astrocytoma | 5 |
|  | Fibrillary astrocytoma | 3 |
|  | Pilocytic astrocytoma | 3 |
|  | Anaplastic astrocytoma | 4 |
|  | Pleomorphic xanthoastrocytoma | 1 |
|  | Subependymal astrocytoma | 1 |
|  | Oligoastrocytoma | 1 |
|  | Oligodendroglioma | 2 |
|  | Atypical oligodendroglioma | 1 |
|  | Anaplastic oligodendroglioma | 1 |
|  | Glioblastoma multiforme | 6 |
|  | Liponeurocytoma | 1 |
|  | Central neurocytoma | 1 |
|  | Dysplastic gangliocytoma | 1 |
|  | Neuroglial cyst* | 2 |
| **Tumors of the sellar region** | Pituitary adenoma (hormonally inactive) | 8 |
|  | Somatotropinoma | 1 |
|  | Prolactinoma | 2 |
| **Embryonal tumors** | Vestibular schwannoma | 5 |
|  | Pineocytoma | 1 |
| **Ependymal tumors** | Ependymoma | 2 |
| **Mesenchymal tumors** | Hemangioblastoma | 1 |
|  | Chondroma | 1 |
| **Metastases (primary tumor)** | Uterus | 1 |
|  | Breast | 2 |
|  | Spinal cord | 1 |
|  | Stomach | 1 |
|  | Colon | 2 |
|  | Lung | 2 |
| **Other neoplasms** | Cavernoma (vascular malformation) | 1 |
|  | Atheroma (cutaneous lesion) | 1 |
|  | Hygroma (fluid collection) | 1 |
